# Assessing Different Causes of Crown-of-Thorns Starfish Outbreaks and Appropriate Responses for Management on the Great Barrier Reef

**DOI:** 10.1371/journal.pone.0169048

**Published:** 2016-12-30

**Authors:** Russell C. Babcock, Jeffrey M. Dambacher, Elisabetta B. Morello, Éva E. Plagányi, Keith R. Hayes, Hugh P. A. Sweatman, Morgan S. Pratchett

**Affiliations:** 1 CSIRO Oceans and Atmosphere, Brisbane, Qld, Australia; 2 School of Plant Biology, University of Western Australia, Crawley, WA, Australia; 3 CSIRO Computational Informatics, Castray Esplanade, Hobart, TAS, Australia; 4 Australian Institute of Marine Science, PMB 3, Townsville MC, Qld, Australia; 5 ARC Centre of Excellence for Coral Reef Studies, James Cook University, Townsville, QLD, Australia; Universita degli Studi di Genova, ITALY

## Abstract

The crown-of-thorns starfish *Acanthaster planci* (COTS) has contributed greatly to declines in coral cover on Australia’s Great Barrier Reef, and remains one of the major acute disturbances on Indo-Pacific coral reefs. Despite uncertainty about the underlying causes of outbreaks and the management responses that might address them, few studies have critically and directly compared competing hypotheses. This study uses qualitative modelling to compare hypotheses relating to outbreak initiation, explicitly considering the potential role of positive feedbacks, elevated nutrients, and removal of starfish predators by fishing. When nutrients and fishing are considered in isolation, the models indicate that a range of alternative hypotheses are capable of explaining outbreak initiation with similar levels of certainty. The models also suggest that outbreaks may be caused by multiple factors operating simultaneously, rather than by single proximal causes. As the complexity and realism of the models increased, the certainty of outcomes decreased, but key areas that require further research to improve the structure of the models were identified. Nutrient additions were likely to result in outbreaks only when COTS larvae alone benefitted from nutrients. Similarly, the effects of fishing on the decline of corals depended on the complexity of interactions among several categories of fishes. Our work suggests that management approaches which seek to be robust to model structure uncertainty should allow for multiple potential causes of outbreaks. Monitoring programs can provide tests of alternative potential causes of outbreaks if they specifically monitor all key taxa at reefs that are exposed to appropriate combinations of potential causal factors.

## Introduction

Outbreaks of the crown-of-thorns starfish (COTS), *Acanthaster planci*, have been a major concern for managers of Indo-Pacific coral reefs since the first well-documented outbreaks in the 1960s ([1], also reviewed in [2]). The devastating effects of high densities of COTS (e.g. [3, 4]), have motivated significant direct, diver-based control efforts. Since the 1960s, divers have killed or removed more than 17 million COTS from Indo-Pacific coral reefs [5] but, even with sustained effort, direct control methods have often been unable to prevent significant loss of coral [6], and periodic outbreaks of COTS remain a major cause of coral mortality in many locations [7, 8]. More effective long-term management of COTS outbreaks must be based on clear understanding of the underlying causes of outbreaks and how they are initiated [5]. Here for the first time we compare and assess the range of causes and mechanisms, in a rigorous and transparent manner, using qualitative modelling as a way to prioritize research into the origins of COTS outbreaks.

Several hypotheses have been put forward to explain the occurrence of population outbreaks of *Acanthaster* spp. (reviewed by [2, 5, 9], emphasizing either biological traits of COTS such as rapid growth and phenomenal reproductive capacity, [10] that predispose them to major population fluctuations (e.g., ‘natural causes hypothesis’, [11]; ‘adult aggregation hypothesis’, [12]; ‘prey-threshold hypothesis’, [13] or anthropogenic changes in environmental conditions that have eroded normal regulatory processes, leading to largely unbounded population fluctuations (e.g., ‘terrestrial run-off hypothesis’, [14]; ‘predator removal hypothesis’, [15]. The extent to which outbreaks are caused or exacerbated by anthropogenic disturbances (i.e., coastal development and overfishing) has a major bearing on deciding appropriate management responses (e.g. [16], but no single hypothesis has unequivocal and universal support [2, 5]. Many biologists and theoretical ecologists concur that it is unlikely that a single factor explains the diverse incidences of crown-of-thorns outbreaks (reviewed by [9, 17], which range from localized outbreaks on small isolated atolls (e.g., Chagos; [18], Moorea [19]), to chronic, large-scale outbreaks (Japan; [20], Oman; [21]). This is not to say, however, that a single factor (e.g., elevated nutrients [14]), or the life history traits of *Acanthaster* alone [22], could not explain the initiation and spread of COTS outbreaks under some circumstances.

On Australia’s Great Barrier Reef (GBR) there have been four documented waves of COTS outbreaks [5]. The first of the outbreaks was detected in 1962 at Green Island [1], though it is possible that many earlier outbreaks went largely undetected [11, 23]. Since 1962, there have been three additional waves of outbreaks, commencing in 1979, 1993, and 2010. During each of these four well-documented outbreaks, high densities of starfish were first detected on mid-shelf reefs between Cairns and Cooktown (the “initiation box”, [16]) and then propagated southwards in accordance with prevailing hydrodynamic conditions over *ca*. 15 years at rates of approximately 50–100 km·y^-1^ [24–27]. Genetic studies have confirmed that reef-wide outbreaks on the GBR are linked to the initiation of primary outbreaks north of Cairns [28, 29]. Moderate levels of connectivity among reefs on the GBR then facilitate a “travelling wave” of secondary outbreaks away from the area of initiation [30, 31]. It is widely accepted that the immense numbers of larvae produced by high densities of well-fed starfish (i.e., the primary outbreak) will inevitably lead to subsequent secondary) outbreaks on downstream reefs. Therefore understanding the cause(s) of outbreaks, and thereby identifying the most appropriate management responses, requires unequivocal focus on the factors that contribute to the original initiation of distinct outbreaks (e.g., [32]), rather than specific patterns of occurrence for secondary waves of outbreaks. These insights will be valuable in the many areas of the Indo-Pacific region where there are ongoing attempts to understand and manage recent COTS outbreaks that stretch from Oman, through Malaysia to Moorea and north to Okinawa [6, 19, 21, 33, 34].

Paleontological evidence relating to the occurrence of past outbreaks is ambiguous [35, 36], though demographic models of populations of massive corals suggest outbreaks of crown-of-thorns starfish may now be more frequent and more intense than at any stage in the past several hundred years [37]. This in turn suggests that anthropogenic disturbances (e.g., coastal development, poor land-use practices, and overfishing) have directly contributed to the increased incidence and or severity of COTS outbreaks [38, 39]. Alternatively, the increasing incidence and diversity of disturbances, and their cumulative impact on coral reefs, may have undermined the capacity of reef ecosystems to withstand sustained outbreaks of *Acanthaster* spp. [5]. What is certain is that the GBR, and reefs more widely, cannot sustain the levels of disturbance caused by COTS outbreaks since the 1980s [8].

### Elevated nutrients and the initiation of outbreaks on the great barrier reef

Sediment and nutrient levels on the GBR (especially near-shore environments) have increased significantly since European settlement [40], and many authors have suggested enhancement of larval survivorship through nutrient enrichment is the fundamental cause of outbreaks, both on the GBR (e.g., [1, 16, 41]), and elsewhere [14, 42]. Lucas [43] suggested that the amount of phytoplankton required to maintain cultured larvae was much higher than generally occurs within the GBR lagoon, leading him to conclude that outbreaks only occur following major phytoplankton blooms (“larval starvation hypothesis”; [43]). Similarly, Fabricius et al. [16] reported minimal survival of larval *Acanthaster planci* at chlorophyll concentrations below 0.25 μg.l^-1^, whereas larval survival increased approximately eightfold with each doubling of chlorophyll concentrations up to 3.0 μg.l^-1^. Fabricius et al. [16] also argue that elevated nutrients in the GBR lagoon are directly attributable to major flood events. They point out that the region of elevated nutrients only overlaps with mid-shelf reefs, between 14.5°S-17.0°S, and hence it is only here that nutrient concentrations exceed the threshold (>0.25–0.5 μg.l^-1^) necessary for enhanced survivorship of crown-of-thorns larvae, thereby explaining initiation of outbreaks in a relatively discrete area north of Cairns.

The nutrient enrichment hypothesis (also referred to as the larval starvation hypothesis) is one mechanism by which anthropogenic activities may have exacerbated outbreaks of *Acanthaster* spp. (increasing their severity and/ or frequency) over recent decades [38, 39]. There remains however, some controversy as to whether COTS larvae are generally food limited (reviewed by [5]), and while in some cases outbreaks coincide with rainfall and nutrient inputs [22] in others they do not [6]. Moreover, if the productivity of mid-shelf waters on the GBR are consistently below levels (0.25μg.l^-1^) at which there is almost zero survival of COTS larvae (e.g., [16]), it is hard to explain how the southward propagating waves of outbreaks (that subsequently cause widespread devastation) are maintained.

### Overfishing and predatory-release for *Acanthaster* spp.

One of the earliest hypotheses to account for outbreaks of *Acanthaster* spp. was the “predator removal hypothesis” [15], which assumed that populations of crown-of-thorns starfish are normally regulated by high levels of early post-settlement or adult predation [18, 44]. Sustained and ongoing fishing on the GBR has certainly resulted in significant decreases in populations of large predatory reef fishes [45] and other potential predators [15]. Moreover, there is evidence that incidence and/ or severity of outbreaks is highest in areas subject to high levels of fisheries exploitation [46, 47]. On the GBR, Sweatman [47] showed that outbreaks were more than 3 times more likely on reefs subject to fishing, compared to reefs that had been closed to fishing (no-take areas) for at least 5 years. More recent analyses have also shown substantially decreased relative intensities of COTS outbreaks on reefs newly closed to fishing [48].Despite their apparent toxicity, COTS are susceptible to predation, as shown by the high incidence (up to 67%) of sub-lethal injuries [44, 49]. Modelling exercises have also shown this hypothesis to be plausible under certain functional response and density-dependent conditions [44, 50–52]. However, it has proven very difficult to identify either the source of predation mortality [21] and whether it might be due to large fish consuming adult COTS or small fish and benthic invertebrates consuming juvenile COTS [52] or the conditions under which juvenile predation might effectively limit COTS populations. There has also been almost no work assessing the extent to which COTS larvae may be susceptible to predation, especially at the point of settlement.

This paper uses qualitative models to explicitly test and compare alternative hypotheses for the initiation of COTS outbreaks on the GBR, and thereby guide appropriate management responses. Modelling approaches are a useful means for *a priori* assessment of complex and interacting process, and have been used successfully in the past to assess the dynamics of COTS populations including the role of predators in initiating outbreaks [44, 53], and the propagation of waves of outbreaks along the GBR [26, 54]. More recent observations relating to the role of nutrients [16, 38] and of predation [47] in outbreaks of COTS and corals on the GBR mean that it is timely to re-evaluate our conceptual understanding of COTS outbreaks in order to assess differing hypotheses and formulate more effective management strategies. We develop qualitative mathematical models around each of the three broad causal theories above to explain the initiation of outbreaks and to assess a range of theories relating to the facilitation of COTS outbreaks in order to identify (i) dynamics and signals that indicate which mechanisms are plausible in any given situation; and (ii) management responses which are robust to our uncertainty around which mechanisms are the main drivers behind primary COTS outbreaks. The hypotheses being tested are that the certainty of model predictions are affected by i) varying levels of connectivity and positive feedback within COTS population; ii) nutrient enhancement of larval survival of both COTS and other planktotrophic species; iii) removal of predators; iv) variation in both nutrients and predation, where both are likely to affect model predictions.

## Methods

### Qualitative mathematical modeling

We developed qualitative mathematical models of COTS interactions in coral reef ecosystems based on published hypotheses and also on elaborations of these hypotheses that we considered plausible. As these are qualitative models their validity and usefulness largely depends on whether or not they offer directional predictions (e.g. increase in COTS, decrease in coral) consistent with real world observations such as declines in coral cover on the GBR over the past several decades. To be plausible, models must provide a causal representation of the ecological theories and hypothesis on which they are based, but also produce predictions for COTS and coral populations. If plausible models give conflicting predictions, then further research and targeted observations are needed to quantify relationships or refine model structure and ultimately reject inaccurate models.

To minimise model complexity, operational units within the system were considered at the level of functional groups (e.g. predators) rather than as individual species or families. Key pressure and response variables (Table 1) central to each of the alternative hypotheses were portrayed using sign directed digraphs, or signed digraphs, following the methods described in Puccia and Levins [55]. Signed digraph models of ecosystems commonly include trophic interactions; such as in a predator‐prey interaction, where the positive benefit to a predator of consuming a prey represents a rate of birth, and the negative effect to the prey represents a rate of mortality. Signed digraph models can also include different life stages of a population, which is useful for integrating across different habitats used by a species throughout its life history [56]. Here the links represent processes supporting or leading to each life stage, such as rates of fecundity, survival or mortality. Any number of life stage variables can be accommodated, as long as the overall feedback, or matrix determinant, of the expanded set of life stage variables equals the single variable’s self-effect for the population as a whole [57]. Many of the models also include links representing what are called modified interactions [58], which describe environmental or ecological variables that can regulate the intensity of the interaction of other variables—an example is when a predator’s ability to capture juvenile prey can be reduced if the juvenile prey use coral to escape, thus coral structure modifies (suppresses) the predator‐prey interaction. In many instances there is uncertainty in the understanding of relationships, which in this context translates into model structure uncertainty, and leads us to consider a range of models representing different system dynamics through alternative model structures i.e. different links between units within a singed digraph model.

**Table 1:** Definition of functional groups.

Pressure	
Nutrients	Nutrients, principally anthropogenically derived Nitrogen in various forms, arising indirectly through land clearing, and land-use practices that lead to accelerated run-off from wet season rains and unnaturally high levels of nutrients in runoff due to excessive fertilizer application [59, 60]. This process can lead to elevated concentrations of phytoplankton and changes in phytoplankton composition in nearshore and mid-shelf waters affected by runoff following extreme weather events [38]. Effects are reduced in remote or offshore waters (e.g. Cape York, Swains Reefs)
Fishing	Broadly targeted Recreational and Commercial fishing for reef associated predatory fish species, as well as collecting of invertebrates (giant Triton *Charonia tritonis*). Effects reduced in Marine Park no-take zones, and remote areas [45]. Anthropogenic pressures from fishing and land use can also act in combination.
Response	
COTS* larva	The planktotrophic pelagic larval phase of *A*. *planci* (COTS larva) is thought to benefit from terrestrially derived nutrients that are washed into coastal waters by storms, particularly where land is under intensive cultivation and fertilizer application [38].
COTS* juvenile	The juvenile phase of *A*. *planci* (COTS juvenile) is highly cryptic, hiding under corals and in reef interstices, feeding exclusively on crustose coralline algae or CCA [52]. At this size they are vulnerable to a wide range of predators, with small invertebrate predators able to feed only on this stage of COTS [61]. Feeding switches to the coral phase after around 6 months and individuals grow rapidly once this occurs.
COTS* adult	Adult *A*. *planci* (COTS adult) live for several years, spawning at around 2 years old and releasing tens of millions of eggs [62]. Many adult starfish (up to 80%) have evidence of recent injuries, presumably caused by predatory fishes [49], but it is unknown to what proportion are killed outright by predators.
CCA	CCA is important at more than one point in the life history of COTS as well as for other reef invertebrates. CCA is a settlement cue for COTS [63], as well as habitat and food source for juvenile COTS [52].
Invertebrate predators	This functional group (Inverts) consists of small predators and consists of species from diverse groups including decapods crustaceans, polychaetes, gastropod mollusks and flatworms [61]. These groups also have planktotrophic larvae that may benefit from increased phytoplankton concentrations [64–67]
Fish large	These are fish species targeted by commercial and recreational fishers, some of which are recorded to consume COTs adults (reviewed by [5]). These species may also consume juvenile COTS.
Fish small	Numerically abundant generalist carnivores generally too small to be targeted by fishers, but trophically important on reefs (e.g. small labrids, lethrinids, cheilinids) as predators of small invertebrates [68] This may include COTS juveniles [69]. These small fish are potential prey for many of the large fish species listed above thus indirectly affected by fishing and changes in large fish abundance.
Fish non-target	There are certain species of fish that are known at times to feed specifically on COTS adults (e.g. toadfish *Arothron stellatus*, (RCB personal observations), and other pufferfishes and triggerfish [70, 71]. These species are not directly targeted by fishers on the GBR but triggerfish may be captured incidentally and retained [72].
Giant Triton	The gastropod *Charonia tritonis* is a carnivore specializing in echinoderm prey, including *A*. *planci* [1]. Its numbers may have been reduced by collecting throughout the 20^th^ Century although it is now fully protected.

* *COTS*. There are three distinct life history phases in *A*. *planci*, and there are key aspects of potential explanations for outbreaks relating to each of them.

We examine the predicted response of the model systems to a press perturbation, whereby model variables reach a new equilibrium as a result of a sustained change in a rate of growth for one or more variables. In a simple system (e.g. coral as prey of crown-of-thorns starfish predator) the response of one component of the system due to an increase in the growth rate of another is simple and unambiguous, however with larger numbers of components and a greater number of indirect interactions in a system the complexity of indirect interactions and their potential to counter the influence of direct interactions increases. Where all pathways of direct and indirect interaction are of the same sign then the result of a press perturbation is unambiguous, with positive effects resulting in an increase in the equilibrium level of a variable, negative a decrease, or no change when there are no pathways leading to a variable from the source of the perturbation. However, where there are both positive and negative effects the predicted sign of the response is ambiguous in terms of whether there would be an increase or decrease. By considering the relative balance of positive vs. negative effects in a response prediction, one can assign a probability for sign determinacy to predictions base on numerical simulations [73, 74]. For example, where there is a three-to-one ratio of positive to negative effects in a response prediction, then the probability of a positive response in numerical simulations generally exceeds 90%, with the magnitude of the negative effects overwhelming positive effects occurring less than 10% of the time [67, 68].

We used this approach to assign a level of confidence in the predicted responses of components of the different models due to the pressures of increased nutrients and overfishing. We used a threshold value of 0.8 probability to distinguish high from low levels of sign determinacy for response predictions. Where sign determinacy is 1.0, then the sign of the predicted response is completely determined and is reported as, either positive “**+**”,negative “**-**“, or zero “0”. Where sign determinacy is less than 1.0 but greater than or equal to 0.8, the predicted response is allocated a sign encased by parentheses–i.e. either positive (+) or negative (-); below this threshold the predicted response is denoted by a question mark “?”.

Nutrients and fishing are pressures on natural systems that are represented in the models as press perturbations. These are examined both independently and in combination, across a range of model configurations that move progressively from simple to more complex, in a series of “pressure scenarios” [75]. The importance of life history traits in the model scenarios is examined by varying the feedback within COTS populations. Importantly, the factors considered here are present in different combinations in different parts of the GBR, for example fished and unfished zones in the central GBR region are periodically exposed to high and low nutrient levels depending on seasonal and inter-annual rainfall variations; while green zones in offshore northern GBR reefs do not experience high fishing pressure or nutrient inputs from runoff. We were therefore able to examine hypotheses involving different combinations of pressures that are appropriate for different zones and regions of the GBR, and which may ultimately be testable through systematic long-term observation and targeted reef monitoring programs.

## Results

**Fig 1:**
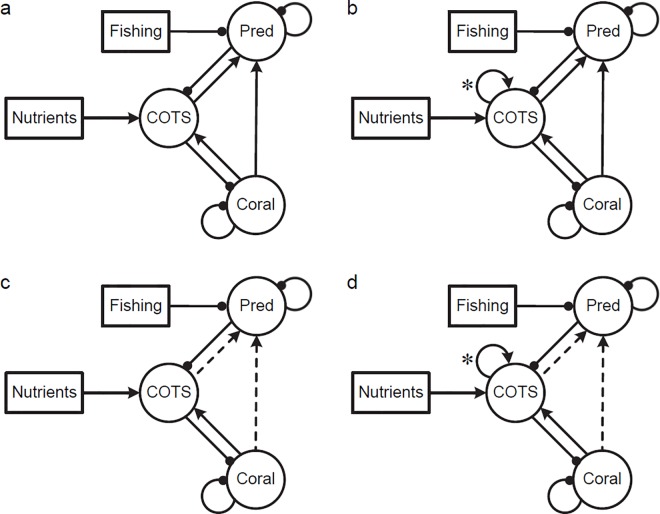
Qualitative model to assess the relative impacts of nutrients and predation on COTS populations. COTS outbreak overview, with human activities leading to increased nutrients (**Nutrients**) and fishing mortality (**Fishing**) of COTS predators (**Pred**). (a) No self-regulating feedback on COTS, with predator benefitting from COTS. (b) Positive self-regulating feedback on COTS, with predator benefiting from COTS (c) No self-regulating feedback on COTS with predators not deriving significant benefit from consumption of COTS (d) Positive self-regulating feedback on COTS with predators not deriving significant benefit from consumption of COTS. Rectangles denote external pressures, circles denote biological system components. New elements of the model structure introduced from one model to the next are denoted by *. Where elements have been removed they are depicted by a dashed line.

We present results from the model variations we consider most informative, starting from the simplest and adding complexity in several stages. The models are:

*Model 1*. A most simple view of the system including both nutrient and predator hypotheses (Fig 1), both of which are influenced by humans and in turn affect COTS, and hence ultimately influence coral cover. Various configurations of this model assess the relative impacts of nutrients and predation on COTS populations where there are varying levels of density-dependent feedbacks within COTS populations, and varying levels of dependence by predators on corals (as habitat) and on COTS (as prey). COTS populations are examined without self-regulating feedback (Fig 1A and 1C) and with positive self-regulating feedback (Allee effects) to reflect the natural causes hypothesis (Fig 1B and 1D) across multiple generations. The positive feedback in models 1b and 1d represents situations such as where numbers of starfish may build up on a reef through self-seeding [76], and then increase rapidly (outbreak) when threshold densities for successful external fertilization are exceeded [77]. The potential role of coral in providing habitat for predators and thereby providing a level of positive feedback on COTS populations is explored in models 1a and 1b. Two classes of COTS predators are distinguished: “specialists” whose population dynamics are strongly linked to the availability of COTS prey (e.g. Triton; Fig 1A and 1B) and “generalists” that only feed incidentally on COTS and so affect starfish numbers but the population dynamics of the predator are largely independent of starfish numbers (Fig 1C and 1D).

In all of the models examined (1a-1d) the sign (positive or negative effect) of the effect of human activities on corals and COTS was the same with COTS increasing and coral decreasing with increases in nutrients and/ or increases in fishing. These outcomes were predicted with uniformly high levels of certainty (Table 2, models 1a-1d) across all models and all combinations of press perturbations.

**Table 2:** Qualitative response predictions for press perturbations to models in Figs 1–5.

	COTS			Predator			Coral			COTS L			COTS J			COTS A			CCA			Invert			Fish S			Fish L			Triton			Fish N		
Model	N	F	N&F	N	F	N&F	N	F	N&F	N	F	N&F	N	F	N&F	N	F	N&F	N	F	N&F	N	F	N&F	N	F	N&F	N	F	N&F	N	F	N&F	N	F	N&F
1a	**+**	**+**	**+**	**?**	**-**	**(-)**	**-**	**-**	**-**																											
1b	**+**	**+**	**+**	**?**	**?**	**?**	**-**	**-**	**-**																											
1c	**+**	**+**	**+**	**0**	**-**	**-**	**-**	**-**	**-**																											
1d	**+**	**+**	**+**	**0**	**-**	**-**	**-**	**-**	**-**																											
2a							**-**			**+**			**+**			**+**																				
2b							**-**			**+**			**+**			**+**																				
2c							**-**			**+**			**+**			**+**																				
2d							**-**			**+**			**+**			**+**																				
3a							**(+)**			**(+)**			**(-)**			**(-)**			**(-)**			**(+)**														
3b							**(+)**			**+**			**(-)**			**(-)**			**-**			**+**														
3c							**?**			**(+)**			**?**			**?**			**0**			**(+)**														
3d							**?**			**+**			**?**			**?**			**0**			**+**														
4a							**?**	**?**	**?**	**(+)**	**0**	**(+)**	**?**	**(-)**	**?**	**?**	**?**	**?**				**?**	**?**	**?**	**?**	**(+)**	**?**	**(+)**	**(-)**	**?**						
4b							**?**	**?**	**?**	**(+)**	**0**	**(+)**	**?**	**(-)**	**?**	**?**	**?**	**?**				**(+)**	**?**	**?**	**?**	**(+)**	**?**	**(+)**	**(-)**	**?**						
4c							**?**	**?**	**?**	**0**	**0**	**0**	**?**	**(-)**	**?**	**?**	**?**	**?**				**?**	**?**	**?**	**?**	**(+)**	**?**	**?**	**(-)**	**?**						
4d							**(-)**	**?**	**(-)**	**(+)**	**0**	**(+)**	**(+)**	**(-)**	**?**	**(+)**	**?**	**(+)**				**-**	**?**	**?**	**?**	**(+)**	**(+)**	**-**	**(-)**	**(-)**						
5a							**?**	**?**	**?**	**?**	**0**	**?**	**?**	**?**	**?**	**?**	**?**	**?**				**?**	**?**	**?**	**?**	**?**	**?**	**(+)**	**(-)**	**?**	**?**	**?**	**?**	**(-)**	**(+)**	**?**
5b							**?**	**(-)**	**?**	**(+)**	**0**	**(+)**	**?**	**?**	**?**	**?**	**(+)**	**?**				**(+)**	**(-)**	**?**	**(+)**	**?**	**(+)**	**(+)**	**(-)**	**?**	**?**	**(-)**	**?**	**(-)**	**(+)**	**?**
5c							**-**	**(-)**	**(-)**	**(+)**	**0**	**(+)**	**(+)**	**?**	**(+)**	**+**	**(+)**	**(+)**				**-**	**(-)**	**(-)**	**-**	**?**	**?**	**-**	**(-)**	**(-)**	**+**	**(-)**	**?**	**+**	**(+)**	**(+)**
5d							**?**	**?**	**?**	**?**	**0**	**?**	**?**	**?**	**?**	**?**	**?**	**?**				**?**	**?**	**?**	**?**	**?**	**?**	**-**	**(-)**	**(-)**	**?**	**?**	**?**	**+**	**(+)**	**(+)**

Predicted response signs with a probability of 1 are indicated in plain text “+”, “-“, “0”; those with relatively high probability of sign determinacy (≥0.8) are enclosed in parentheses (+) (-); “?” denotes those with a low probability of sign determinacy <0.8.

Press perturbations: N-increase in nutrients, F-increase in fishing mortality, N&F-simultaneous increase in nutrients and fishing mortality.

**Fig 2:**
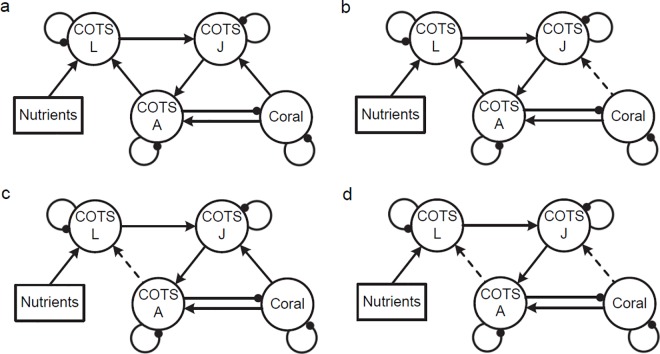
Nutrient input hypothesis for COTS closed and open population. **COTS A**: COTS adult, **COTS J**: COTS juvenile, **COTS L**: COTS larvae. (a) Closed COTS population where coral facilitates COTS juveniles. (b) Closed COTS population with no facilitation of COTS juveniles by corals (COTS juveniles increase with nutrient addition due to the effect of nutrients on COTS larvae therefore positive effects from nutrients flow directly to them) (c) Open COTS population where coral facilitates COTS juveniles (d) Open COTS population with no facilitation of COTS juveniles by corals.

*Model 2*. Effect of nutrients on COTS larvae. These models include multiple COTS life history stages, (adults, juveniles and larvae) and scenarios assuming both closed (or self-seeding) populations (Table 2, Fig 2A and 2B) and open populations (that receive dispersive larvae) (Fig 2C and 2D). The models also allow us to explore the relative benefit of coral to juvenile COTS either as food for emerging juveniles or indirectly through shading and the facilitation of crustose coralline algae (CCA) which is a juvenile food source (Fig 2A and 2C). All COTS populations had negative density-dependent self-regulation. Whether closed (Fig 2A and 2B) or open (Fig 2C and 2D) populations were considered, and regardless of any influence of coral on COTS juveniles, adult COTS populations increased and coral decreased with addition of nutrients. The certainty of these outcomes was high and did not differ among the various models.

**Fig 3:**
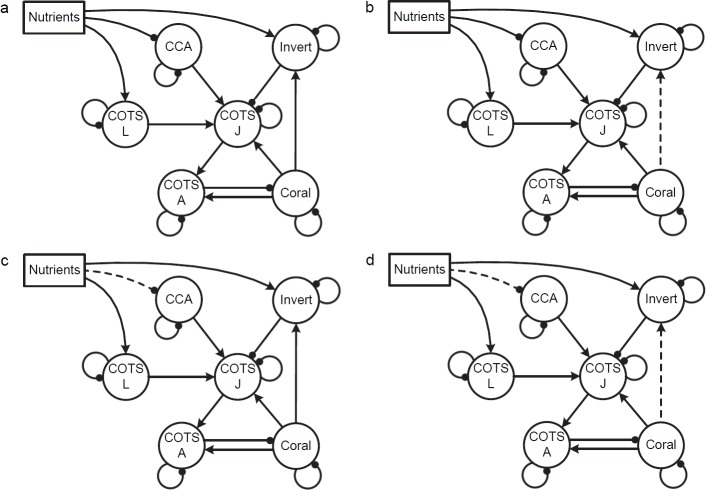
Effects of nutrients on COTS (open population) and reef invertebrates. **Invert:** reef invertebrates, **CCA**: crustose coralline algae, **COTS A**: COTS adult, **COTS J**: COTS juvenile. (a) Coral facilitates invertebrate predators. (b) No facilitation by corals. (c) Coral facilitates invertebrate predators with no effect of nutrients on CCA (d) No facilitation of invertebrates by corals and no effect of nutrients on CCA.

*Model 3*. Effects of nutrients on planktotrophic larval assemblages. Nutrients may affect not only COTS larvae but also the larvae of invertebrate species that prey on juvenile COTS. The larvae of COTS and invertebrates may also be assumed to benefit from higher levels of phytoplankton. The effects of nutrients on crustose coralline algae (CCA) may also be important since CCA is a settlement cue, habitat and food source for juvenile COTS. In this model, we have assumed that increased levels of nutrients on reefs indirectly inhibit growth of CCA (Fig 3A and 3B) by enhancing growth of other algae [78, 79], as well as cases where nutrient additions had no effect on CCA (Fig 3C and 3D). Invertebrate predators of juvenile COTS [61] are considered both with (Fig 3A and 3C) and without (Fig 3B and 3D) facilitation by corals. The most interesting feature of this set of models is that an increase in nutrients can lead to an increase in coral and a decrease in COTS adults, due to the increase in invertebrate predators (Table 2). The effects of nutrient addition on COTS adults and coral cover varied markedly across the different formulations of the model, however with increases in coral and decreases in COTS predicted with low levels of certainty only if there is a negative impact of nutrients on CCA (Table 2, Fig 3A and 3B). With no effect of nutrients on CCA the combined positive influences of nutrients on COTS larvae and negative impacts of increased predation by invertebrates cancelled each other out and there was an ambiguous prediction for the effect of nutrients on coral and COTS adults (Table 2, Fig 3C and 3D). This was quite in contrast to the outcomes predicted by models 2c and 2d which otherwise produce virtually identical outcomes apart from the influence of invertebrates as predators on COTS juveniles. CCA was not included in subsequent models given that the prediction of increased coral cover and reduced COTS under conditions of nutrient addition are at odds with observations on reefs that appear to be able to support COTS outbreaks.

**Fig 4:**
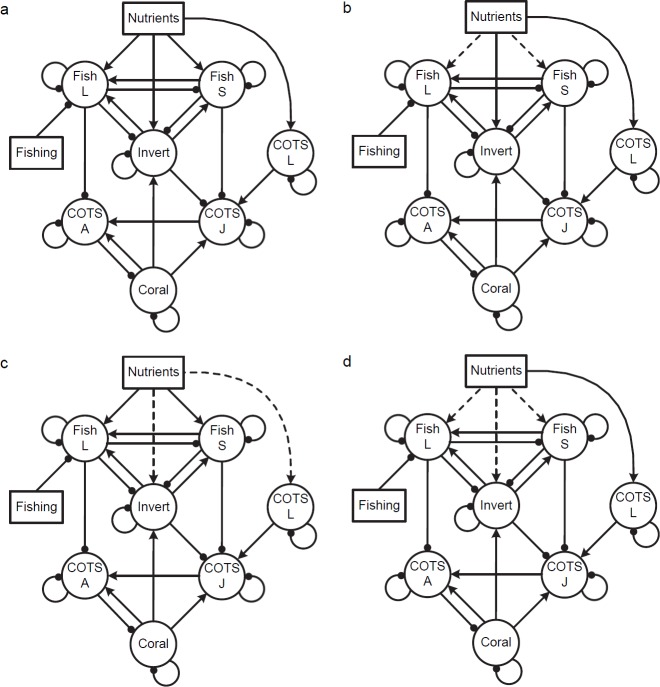
Model of effects of nutrients on a broader range of larval groups. Effects of nutrients (**Nutrients**) on COTS (open population), reef invertebrates (**Invert**), and large (**Fish L**) and small (**Fish S**) predatory fishes; **CCA**: crustose coralline algae, **COTS A**: COTS adult, **COTS J**: COTS juvenile. (a) Nutrients facilitate larvae of invertebrates and fish groups as well as CCA. (b) Nutrients facilitate invertebrates and CCA only. (c) Nutrients facilitate fish but not invertebrates or CCA. (d) Nutrients facilitate CCA only.

*Model 4*. This model considers the effects of nutrients on a broader range of larval groups because some fish populations may also be affected by larval food availability [65] and larval supply [80]. The adults of these fish and invertebrate groups all interact across a range of trophic levels with potential effects on both COTS and corals (Fig 4A–4D). Versions of this model (4a, 4b) show ambiguous effects of nutrients on abundance of COTS adults and coral (Table 2). Model 4d was similar to models 4a-4c, but without the effects of nutrients on predators there was a prediction of increased COTS and reduced corals though not with high levels of certainty (Table 2). With increased fishing pressure alone the model predicted ambiguous effects on coral and COTS adults, essentially due to the three-way interaction among fish and invertebrates whereby any increase in small fish due to release from large fish predation by fishing is counteracted by a decrease in invertebrates. With a combined press on both nutrients and fishing, the results were the same as for a nutrient increase. To sum up, the only conditions under which a COTS outbreak could be unambiguously emulated was if COTS larvae were the only taxon to respond positively to increased nutrients.

**Fig 5:**
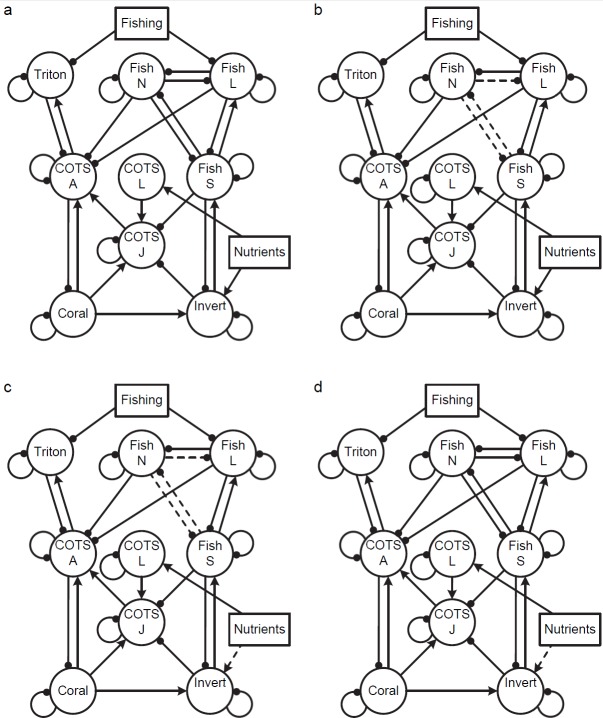
Example of a more complex set of models of COTS -coral interactions. Effects of fishing (**Fishing**) and nutrients (**Nutrients**) on COTS open populations. Predicted responses to press perturbations were examined for each model for Nutrients or Fishing alone, and then Nutrients and Fishing in combination. (a) Nutrients benefit larvae of COTS as well as invertebrates, fish assemblages show high level of interaction through competition and predation. (b) Nutrients benefit larvae of COTS as well as invertebrates, fish assemblages show low levels of interaction (c) Nutrients benefit COTS larvae alone, fish assemblages show low levels of interaction (d) Nutrients benefit COTS larvae alone, fish assemblages show high levels of interaction. **COTS A**: COTS adult, **COTS J**: COTS juvenile, **Fish L**: targeted large fish predators, **Fish N**: non-target fish predators, **Fish S**: targeted small fish predators, **Invert**: invertebrates, **Triton**: Giant Triton. **COTS A**: COTS adult, **COTS J**: COTS juvenile, **Fish L**: targeted large fish predators, **Fish N**: non-target fish predators, **Fish S**: targeted small fish predators, **Invert**: invertebrates, **Triton**: Giant Triton.

*Model 5*. This model examines the effects of both fishing and nutrients on COTS populations in a more complete combination of scenarios with nutrients influencing a range of invertebrate larvae including COTS larvae, and with multiple sources of predation on COTS juveniles and adults. Effects of fishing and predators on COTS are explored by allowing adult and juvenile COTS to be preyed upon by a range of target and non-target fish species, as well as by predators that significantly benefit from COTS consumption (both fish and invertebrate). Predators (large fish, non-target fish and small fish) may act on either adults or juveniles, with different suites of predators involved at the various life history stages (Fig 5A–5D). Varying levels of interactions between large fish, non-target fish species and small fish predators are assessed; models 5a and 5d include a full range of interactions (large fish compete with non-target fish and with smaller fish prey species), in models 5b and 5c non-target fish species do not interact with small fish.

This model includes the positive effects of nutrients on both COTS and invertebrate larval assemblages (Fig 5A and 5B) and on COTS larvae alone (Fig 5C and 5D). When nutrients influence COTS larvae as well as invertebrate larvae and there is a full range of interactions among fish groups (Fig 5A) outcomes are highly uncertain and no clear effects are predicted in corals or COTS adults (Table 2). As noted previously, where nutrients benefit both COTS and their invertebrate predators, positive and negative effects on COTS may balance out. In the case of fishing, a full range of interactions means that reductions in targeted large fish can release other fish predators of COTS either directly or indirectly (non-target fish). While fishing releases small fish from predation, it indirectly increases non-target fish competition with small fish. The result of these interactions is no net effect on invertebrates and reduced positive fishing impacts on COTS. Where nutrient effects remain balanced and affect a range of invertebrate larvae but the range of fish interactions is reduced (Fig 5B), only fishing resulted in increases in COTS and decreases in coral, though with some uncertainty in both cases (Table 2).

If the positive effect of nutrients act only on COTS and where fish interactions are reduced (Fig 5C), the model predicts adult COTS populations will increase and corals decrease in all scenarios, though this is less certain for the effects of fishing, either alone or in combination with nutrients (Table 2). With only COTS larvae benefitting from nutrients, and with a full range of interactions among fish (Fig 5D), the outcomes of the model were highly uncertain for COTS and Corals under all scenarios (Table 2). In this case the uncertainty of outcomes introduced by the large number of interactions among fish counterbalanced the direct effects of nutrients to COTS larvae. To summarise, the level of interactions among fish groups in the model strongly affected the certainty of predicted outcomes, regardless of the mechanisms of nutrient effects in the models, with clear predictions possible only where interactions among fish groups were restricted.

## Discussion

The qualitative modelling results indicate that elevated nutrients and removal of key predators (through overfishing) have the potential to cause or exacerbate COTS outbreaks, leading to increases in COTS populations and reductions in coral. In simplified models, outbreaks are predicted with high levels of certainty under conditions of nutrient addition, increases in fishing, or a combination of the two factors. In the most simple models, altering fishing pressure on COTS predators, and hence the rate of predation on adult COTS, is just as likely to produce changes in the abundance of COTS as changing nutrient regimes. These models support the assertion that there can be multiple and varied causes of outbreaks. This assertion is also supported by observations such as the persistence of COTS outbreaks in the Swains Reefs in the southern GBR that are remote from terrestrial nutrient sources, and the reduced frequency of outbreaks on reefs with no-take zoning in the Cairns and central sections of the GBR [47]. In more complex models, with a larger number of interacting functional groups, outcomes are similar under some sets of circumstances, but they are less certain. This is true even where simultaneous pressures from both nutrients and fishing are simulated. This highlights the inherent difficulty in arriving at a clear understanding of the causes underlying COTS outbreaks and formulating effective management approaches.

### Nutrients

The simplest models clearly show the potential for sustained increases in Nutrients to cause COTS populations to increase in both closed (self-seeded) and open populations. Our models show that if nutrient additions have negative impact on COTS habitat quality (CCA), or enhance the abundance of larvae of other species (competitors and predators) that interact with COTS, then the positive effects of nutrients on COTS populations may be neutral, or less certain. In fact, among all the models examined, nutrient additions only resulted in increases in COTS adults where COTS larvae were the only taxon to benefit from added nutrients. This is because the effects of increased abundance of invertebrate predators on COTS juveniles may be large, as shown experimentally [61]. Unfortunately without knowing the exact nature of these invertebrate COTS predators, and how they may also respond to nutrient levels, and in the absence of routine large-scale monitoring of them, it is impossible to further investigate this hypothesis empirically. At the same time, the possibility that such alternative mechanisms may control COTS outbreaks highlights the dangers of ignoring uncertainty in model structure and of designing monitoring (and management) programs around single-cause hypotheses. There is significant uncertainty concerning both the effects of nutrient inputs on overall plankton assemblages in GBR waters. Similarly while there are in vitro studies of the nutrition of COTS larvae there is no evidence for food limitation in wild COTS larvae such has been demonstrated in other echinoderm larvae [81]. Our results suggest that ignoring this uncertainty can lead to erroneous conclusions about causative mechanisms in ecological systems, a concern also raised in relation to COTS management in other parts of the Indo-Pacific [33].

Models that combine the effects of nutrients with fishing effects show that while small fish predators of COTS juveniles may increase due to release from predators (due to fishing), this may not translate to an effect of fishing on juvenile COTS numbers, mainly because both large and small fishes may prey on juvenile COTS. In these cases the qualitative modelling approach has limitations, since the outcomes of models will depend more on the level or intensity of interaction rather than simply the presence or absence of interactions. Quantitative and dynamic modelling approaches such as Models of Intermediate Complexity for Ecosystems (MICE) are required to further assess such scenarios (e.g. [82]).

### Predation

Our models assume that predators can control COTS populations, as has been predicted independently in previous modelling efforts aimed at understanding COTS outbreaks [44, 51]. Highly simplified models that examine the effects of fishing, either alone or in combination with added nutrients, predict that fishing will have clear effects on both COTS adults and corals with high levels of certainty. In more complex model scenarios where predators interact with each other as well as with COTS, predicted outcomes are far less clear. Sustained increases in fishing pressure result in influences on COTS and coral populations only where there are low levels of interaction among predatory fishes. When fishing pressures are combined with added nutrients in more complex models, the pattern varies somewhat depending on the groups affected by nutrients but remains largely influenced by the interactions among fish predator groups. There is much uncertainty around the potential impacts of changes in either nutrient inputs or in fishing. In part, this is because the exact nature of the predators and their interactions with COTS remains poorly known, so the models cannot be specified more definitively. Again we are hampered here because we lack information on many potentially relevant functional groups that could enable us to distinguish among the competing explanations for the initiation of outbreaks.

There is indirect evidence that predation plays a role in COTS population dynamics since fewer outbreaks have been reported on reefs with reduced fishing pressure [46, 47, 48], but the mechanistic basis for this link is poorly understood, in so much as we do not know which species are key predators on COTS, especially COTS larvae and newly settled individuals. Moreover, these purported links are based on the level of fishing pressure, rather than explicit abundance of potential predatory species. Until more light is shed on the process (or processes) of predation, the ability of managers to use natural feedback mechanisms within ecosystems to better manage coral reefs will be limited. Evidence of systematic predation on COTS by fish also remains scarce [21], however, managing the local abundance of predatory species (especially if they are fisheries target species) can apparently reduce the impacts of COTS on coral reefs relatively rapidly (e.g. less than 15 yrs, c.f. [47, 48]) compared with lowering nutrient concentrations through managing land use.

### Implications for management

Our results cannot eliminate either increased nutrients or increased fishing, or a combination of these factors, as viable mechanisms to explain primary COTS outbreaks. These four models are very simple and general in their nature and, while they have relatively high levels of certainty, they do not represent many of the processes that are known to influence COTS populations on reefs. They do however, support the assertion that primary outbreaks on the GBR can be caused by multiple factors including a) nutrients: enhanced larval survival and recruitment influenced by water column biogeochemical properties (bottom up forcing); b), effects of fishing: top-down outbreak control by predation occurring under different density-dependence situations and through more or less complex trophic cascades [17, 31, 46, 52]. They also suggest that Allee effects or positive feedback within closed populations in regions of favourable local oceanography and connectivity (e.g. GBR between Lizard Island and Cairns) do not appear to be essential for outbreaks although the possibility remains that such effects may be influential in a quantitative sense, particularly in meta-population scenarios.

More complex qualitative models of the COTS-Coral system provide lower levels of certainty; if we are to improve the levels of certainty in our general understanding and modelling efforts, we must be able to identify those components of the system that are most important and eliminate those that are less relevant. Many different trophic interactions may influence predation on COTS, some of which we have represented in models here. However at present we still require more information in order to identify the minimum set of components that need to be included in order for a model to be useful. Models involving aspects of the natural causes and nutrient hypotheses are heavily reliant on recruitment, a process that is notoriously variable in echinoderm populations [83] including COTS. All these processes may interact in non-linear ways, for example predation is likely to be disproportionately important at low densities due to Type II or Type III functional responses, the effect of which may be further enhanced by Allee effects at low densities [84] as has been previously documented in COTS populations [46]. In this scenario COTS populations may be most effectively controlled by natural processes, such as infrequent predation on low density populations, when no outbreaks are evident. It is vital for the long term survival of the reefs on the GBR that the causes of outbreaks are understood in detail, so that more effective measures to manage coral reef ecosystems can be taken. It is also important that observations that are relevant to these mechanisms are monitored routinely.

In order to have the best chance of providing the information needed to effectively manage COTS on the GBR, conclusions from this modelling approach need to be explicitly tested with targeted research and an integrated monitoring program. Our models suggest that a management strategy that targets any single cause of COTS outbreaks may be based on an uncertain premise, and risks neglecting other potentially useful approaches. Modelling has shown that predation is unlikely to control COTS once outbreak populations have become established, and that if predation can control populations it must take place before this point in the population cycle [44], either on adults at low densities (below reproductive thresholds—Allee effect) or on juveniles before they emerge into the coral-feeding phase [82]. At this point, qualitative models cannot differentiate strong from weak Allee effects or the interaction between the strength of the Allee effect and mild or strong predation pressure. A better understanding of dispersal and recruitment dynamics, as well as the predators of adult starfish on reefs in outbreak source areas, is required to better assess the potential for pre-outbreak control of COTS populations. Ideally, observations on reefs with a range of predator densities (e.g. fished and no-take reefs) would be required including measurements of abundances across interacting trophic groups.

While monitoring of the effectiveness of no-take zones on the GBR has provided valuable insights into the role of fishing and predation in COTS outbreaks, modelling has highlighted some potentially important gaps in ongoing monitoring efforts. Monitoring can only be used to assess the range of competing hypotheses outlined here if it provides information on all relevant trophic groups. Monitoring on the GBR does specifically compare fished and unfished reefs and provides a strong starting point for ongoing hypothesis testing and adaptive management, but not all relevant trophic groups are currently part of this routine monitoring. Overreliance on actions such as small-scale control measures and changing land use and nutrient inputs may be less effective than predicted if (i) the role of other factors such as predation turns out to be more important than runoff, and/or (ii) it takes too long to turn around biogeochemical cycling and the nutrient regime in GBR catchments and lagoon sediments. Studies of no-take areas on the GBR [47, 48] suggest management of fishing pressure may be successful in controlling COTS within relatively short timeframes, while significant uncertainty remains around how successful land-use management in catchments will be in changing nutrient status of the GBR lagoon, and what is a realistic time scale for change [85]. Consequently, a disproportionate focus on reducing nutrient concentrations, while likely to be generally beneficial for ecosystem health and resilience, is not necessarily the only way to ameliorate future COTS outbreaks.

Active control efforts are being advocated for COTS on the GBR, and indeed some spatially-limited efforts are being conducted in the outbreak initiation zone [5], however long term abatement of COTS outbreaks must ultimately be achieved through homeostatic mechanisms within the GBR ecosystem itself. That is, assuming that the current frequency and intensity of outbreaks is unnatural, as seems to be the case given the declines in coral cover over the past several decades [8]. Considering there are likely to be multiple causes of COTS outbreaks, research to better understand these causes and mechanisms, and to implement effective management measures, must also be multifaceted.

Ongoing research to identify or eliminate, and to quantify, key linkages in the COTS-coral ecosystems of the GBR are clearly required if we are to identify management actions that are most likely to be successful with any degree of certainty. This has long been recognized, but the effort and resources devoted to it have been sporadic to say the least, disappearing and reappearing with the cycles of outbreaks on the GBR (albeit with a small time-lag). The fact that coral cover on the GBR continues to decline dictates that these efforts must not only be renewed but sustained if we are to reduce the frequency of outbreaks on the GBR and in other parts of the Indo-Pacific region. This can best be done if we understand the processes that prevent primary outbreaks and maintain COTS populations at low densities, something that is only possible prior to the initiation of primary outbreaks, not during waves of secondary outbreaks [44, 50].

We recommend that ongoing management of COTS should adopt an approach based on the twin approaches of strategic use of no-take zones (or reducing overall fishing pressure) as well as reducing nutrient inputs. In order to ultimately evaluate the relative roles of these factors in causing more frequent and intense outbreaks of COTS, we also recommend that measurements of all key functional groups modeled here be incorporated into an integrated monitoring program for the GBR. This would consist of a structured set of observations that contrasts levels of terrestrial nutrient inputs, fishing pressure, and connectivity, replicated across the extent of the GBR. Such a program will address a multitude of other management needs, but its potential benefits in terms of informing our understanding of the COTS phenomenon on the GBR will be fully realized only if it is explicitly structured in a hypothesis testing framework. This in turn has important implications for the prevention and management of COTS outbreaks throughout the Indo-Pacific, particularly as monitoring data are scarce in many of these regions.
